# Effectiveness of a Pilot Partner Notification Program for New HIV Cases in Barcelona, Spain

**DOI:** 10.1371/journal.pone.0121536

**Published:** 2015-04-07

**Authors:** Patricia Garcia de Olalla, Ema Molas, María Jesús Barberà, Silvia Martín, Encarnació Arellano, Mercè Gosch, Pilar Saladie, Teresa Carbonell, Hernando Knobel, Elia Diez, Joan A Caylà

**Affiliations:** 1 EpidemiologyService,Agència de Salut Pública de Barcelona, Barcelona, Spain; 2 Internal Medicine-InfectiousDiseases,University Hospital del Mar, Barcelona, Spain; 3 Sexually Transmitted Infections Unit, University Hospital Valld’Hebron, Barcelona, Spain; 4 Preventive Interventions and Programs Service,Agència de SalutPública de Barcelona, Barcelona, Spain; 5 Biomedical Research Consortium of the Epidemiology and Public Health Network (CIBERESP), Barcelona, Spain; David Geffen School of Medicine at UCLA, UNITED STATES

## Abstract

**Background:**

An estimated 30% of HIV cases in the European Union are not aware of their serological status. This study aimed to assess the effectiveness of a pilot HIV partner notification program.

**Methods:**

HIV cases diagnosed between January 2012 and June 2013 at two healthcare settings in Barcelona were invited to participate in a prospective survey. We identified process and outcome measures to evaluate this partner notification program, including the number of partners identified per interviewed index case, the proportion of partners tested for HIV as a result of the partner notification, and the proportion of new HIV diagnoses among their sex or needle-sharing partners.

**Results:**

Of the 125 index cases contacted, 108 (86.4%) agreed to provide information about partners. A total of 199 sexual partners were identified (1.8 partners per interviewed index case). HIV outcome was already known for 58 partners (70.7% were known to be HIV-positive), 141 partners were tested as result of partner notification, and 26 were newly diagnosed with HIV. The case-finding effectiveness of the program was 18.4%.

**Conclusion:**

This pilot program provides evidence of the effectiveness of a partner notification program implemented in healthcare settings. This active partner notification program was feasible, acceptable to the user, and identified a high proportion of HIV-infected patients previously unaware of their status.

## Introduction

It is estimated that 15–38% of cases of HIV infection in the European Union are diagnosed late, and 30% of infected individuals are not aware of their serological status [1,2]. Contact tracing is one of the principal activities of public health, and is crucial for the control of sexually transmitted infections (STI). Partner notification (PN) of people infected with HIV is considered a key strategy for promoting diagnosis and early treatment, thereby reducing HIV transmission at the community level [3]. It also presents the possibility of offering preventive treatment to people assessed in the first hours after exposure [4], as well as allowing exposed individuals to exercise their right to know their risk [5,6]. These interventions have been shown to be cost-effective, and are widely accepted by the beneficiaries of these programs [7].

There are ethical, clinical and epidemiological reasons to justify the incorporation of HIV contact notification and evaluation as a regular part of prevention programs for public health, as for other infections [8]. However, there is considerable variation between European countries in the way in which partner notification is implemented, and this variation is driven by factors such as differences in laws, policies, regulations and clinical guidelines [9,10].

While PN can be an essential tool for controlling HIV transmission, there are still no official recommendations on implementing HIV PN in many countries. The potential of PN for HIV prevention has been ignored, and little research has been carried out in Spain on the effectiveness of HIV PN in detecting undiagnosed HIV [11].

The aim of this study was to assess the feasibility and effectiveness of an HIV PN pilot program in identifying and locating people who are unaware of their HIV infection at two healthcare centres in a large European city.

## Methods

### Recruitment and Data Collection

Over a 10-month period (September 2012–June 2013), all cases of HIV diagnosed between January 2012 and June 2013 and managed for the first time in one of the two participating healthcare centres (Hospital HIV unit and STI Primary Care Centre) were invited to participate as an *"index case"* in a prospective PN survey by providing contact information for their sexual or drug injecting contacts during the previous 12 months before HIV diagnosis. In line with current practice, HIV-infected patients were referred by their attending physician to a public health worker from the local health department (PHW) specifically trained for this intervention. Physicians were also responsible for informing all recruited patients (index cases) about the study, and obtaining their consent to participate. The program includes a protocol for rapid detection of HIV status.

PHW conducted interviews with the index cases using standardized paper forms. Data on demographics characteristic (gender, age, country of birth, and educational level), HIV exposure category(injecting drug users [IDU], sex between men [MSM],heterosexuals [HTS], and injecting drug users and sex between men [MSM*IDU]), clinical data (index cases were classified according to the HIV CDC classification: clinical category A comprised asymptomatic acute or primary HIV infection or persistent generalized lymphadenopathy; clinical category B comprised symptomatic conditions that were not included in clinical categories A or C but were attributed to a cell-mediated immunity defect; and clinical category C comprised AIDS-defining conditions) [12], and CD4 cell count at diagnosis (cell/mm3) were recorded.

HIV risk behaviour factors were classified according to the patient’s self-reported sexual orientation. Index cases were asked to provide the number, gender, names and location information of their partners. Clinical information was collected from the medical records. Participating index cases could choose to inform personally their partners about their exposure (patient referral), have their partners notified by the PHW (provider referral), or a combination of these referral methods (index case notifies some partners, and agrees to have the PHW notify others). Partners located by provider referral were interviewed and offered free HIV rapid testing. Index cases who chose patient referral method were contacted by telephone to know HIV results of their partners. The outcomes of HIV testing for partners informed by patient referral were obtained from their index cases, and were not confirmed by PHW. However, the outcomes of HIV testing for partners who were informed by provider referral were verified by the PHW.

### Statistical Analysis

A descriptive analysis of the epidemiological characteristics of the HIV index case was carried out. Comparisons were made by contrasting the group of Index cases who provided partner information to index cases who did not provide partner information; and across the three groups according to PN method (patient referral, provider referral and combined). Categorical variables were compared using *χ* ^2^ test. For each comparison, the univariate odds ratio (OR), 95% confidence interval (CI) and p-value for statistical significance were computed. Fisher's exact test were used when there were some cells with expected value <5. Continuous variables were compared using the Mann-Whitney U test or the Kruskal-Wallis test. We also calculated the number of partners identified per interviewed index case; the number of partners who underwent HIV testing as result of the PN program; the total prevalence among identified contacts (known HIV-positive partners plus newly diagnosed HIV cases divided by the number of contacts identified), and the number of index cases needed to be interviewed (NNTI) to identify one unknown HIV-infected person [13]. The case-finding effectiveness of the study was calculated by dividing the total number of newly diagnosed partners by the total number of partners tested for HIV as result of the PN program.

In order to make comparisons between groups when a variable had more than two categories, and some of them had small numbers (less than three), we pooled these categories together. HIV risk behaviour variables were classified into the following mutually exclusive categories: men who have sex with men (MSM), and heterosexual (HTS). MSM or HTS patients that used injected drugs were classified as MSM or HTS, respectively if they denied needle-sharing partners, and bisexuals were classified as MSM.

All analyses were conducted using SPSS v18 (SPSS Inc., Chicago, Illinois USA).

### Ethical considerations

Participating centres obtained ethical approval from their institutional committee *“ComitéEtico de InvestigaciónClínicaParc de Salut MAR”*, Mar Hospital, Barcelona (Spain). Whether or not patients consented to participate were recorded in an anonymized list of index cases eligible for the study. Only verbal informed consent was provided in compliance with Article 13 of the law 67/2010 (25th May 2010) of the Health Department of Generalitat de Catalunya. HIV is a mandatory notifiable infection and PN is a requirement for health professionals. The ethical committee approved of this consent procedure. Data were managed in a strictly confidential manner according to the ethical principles of the Helsinki Declaration of 1964 revised by the World Medical Organization in Edinburgh, 2000 and Law 15/1999 of Data Protection in Spain.

## Results

### Characteristics of the index patients

Out of the 131 newly diagnosed HIV index cases eligible for inclusion, a total of 125 were contacted and offered partner notification. 96.0% (120/125) were men, 87.5% of whom (105/120) were MSM, with a median age of 34 years (range: 19–69, n = 125), and 51.2% (64/125) born in Spain. 47.2% of index cases had been tested through periodic screening, 17.6% because they had symptoms of STI, 14.4% because they had possible HIV-related symptoms, 8.0% because they had a regular HIV-infected partner, 6.0% for other causes, and no information was available for 6.8%. On initial clinical examination, 85% of index cases met the criteria for category A of the CDC classification of HIV disease, 5% for category B, and 10% for category C. At the time of diagnosis, 42.4% (53 of 125) of index cases were in a current regular relationship. 12.8% (16 of 125) of the index cases contacted refused to provide information about their partners. No statistically significant differences in terms of sex, age, place of birth, and transmission category between those who agreed to participate in the study and those who refused were observed.

Among the 109 index cases who gave consent to participate in the study; 96.3% were men, 28% were less than 30 years old, most were MSM (83.3%), 55.3% were born in Spain, and 86.1% had casual sexual partner(s). 86 (79.6%) provided information for locating their partners. Table 1 shows the characteristics of the index cases according to whether they provided or not information for tracing partners. These two groups did not differ significantly in terms of age, place of birth, transmission category and number of partners notified. One individual who denied having had sexual or injection contacts was excluded from the analysis.

**Table 1:** Characteristics of the 108 index cases who gave consent to participate in the study by providing partner information. Barcelona, 2012–13.

Variables		Index cases who provided information for tracing partners	Index cases who did *not* provide information for tracing partners	Total
		N = 86 (%)	N = 22 (%)	N = 108 (%)
Gender	Male	82 (95.3)	22 (100)	104 (96.3)
	Female	4 (4.7)		4 (3.7)
Median age (range) in years		33.50 (19–69)	35 (21–60)	34 (19–69)
Place of birth	Spain	46 (53.5)	11 (50.0)	57 (52.8)
	Western Europe	9 (10.5)	2 (9.1)	11 (10.2)
	Latin America	24 (27.9)	3 (13.6)	27 (25.0)
	North Africa	2 (2.3)	2 (9.1)	4 (3.7)
	Eastern Europe	4 (4.7)	3 (13.6)	7 (6.5)
	Other	1 (1.2)	1 (4.5)	2 (1.9)
Educational level	No formal education	2 (2.3)	3 (13.6)	5 (4.6)
	Primary	12 (14.0)	5 (22.7)	17 (15.7)
	Secondary	40 (46.5)	8 (36.4)	48 (44.4)
	University	32 (37.2)	5 (22.7)	37 (34.3)
	Missing		1 (4.5)	1 (0.9)
Transmission category	MSM	75 (87.2)	15 (68.2)	90 (83.3)
	HTS	10 (11.6)	4 (18.2)	14 (13.0)
	MSM and IDU	1 (1.2)	1 (4.5)	2 (1.9)
	HTS and IDU	0	2 (9.1)	2 (1.9)
CD4 cell count at diagnosis (cell/mm^3^)	<350	30 (34.9)	11 (50.0)	41 (38.0)
	350–500	23 (26.7)	3 (13.6)	26 (24.1)
	>500	33 (38.4)	8 (36.4)	41 (38.0)
Mean number of partners (range)		20 (1–360)	13 (1–84)	19 (1–360)

Men who have sex with men (MSM), injecting drug use (IDU), and heterosexual (HTS).

### PN outcomes

108 index cases had one or more sexual contacts in the previous 12 months before HIV diagnosis, reporting 2,050 sexual partners in total. Partners were predominantly male (96.5%) and mostly anonymous. Overall, 199 (9.7%) of these reported partners were identified (1.8 partners identified per index case interviewed).

Table 2 shows the characteristics of index cases according to partner notification method. Thus69.7% (60) of the index cases chose the patient referral approach to inform their contacts, 14.0% (12) the provider referral approach and 16.3% (14) the combined approach. No significant differences were observed in terms ofage, place of birth, and transmission category across the three groups according to PN method.

**Table 2:** Characteristics of index cases according to partner notification method. Barcelona, 2012–13.

Variables		Patient referral n = 60	Provider referral n = 12	Combined approach n = 14	Total
					N = 86
Gender	Male	58 (96.7)	11 (91.7)	13 (92.9)	82 (95.3)
Median age (range) in years		34.4 (19–69)	35.3 (27–44)	32 (22–57)	
Place of birth	Spain	33 (55.0)	5 (41.7)	8 (57.1)	46 (53.5)
	Western Europe	6 (10.0)	1 (8.3)	2 (14.3)	9 (10.5)
	Latin America	19 (31.7)	3 (25.0)	2 (14.3)	24 (27.9)
	North Africa	1 (1.7)	1 (8.3)		2 (2.3)
	Eastern Europe	1 (1.7)	1 (8.3)	2 (14.3)	4 (4.7)
	Other		1 (8.3)		1 (1.2)
Educational level	No formal education	2 (3.3)			2 (2.3)
	Primary	7 (11.7)	3 (25.0)	2 (14.3)	12 (14.0)
	Secondary	29 (48.3)	5 (41.7)	6 (42.9)	40 (46.5)
	University	22 (36.7)	4 (33.3)	6 (42.9)	32 (37.2)
Transmission category	MSM	55 (91.6)	9 (75.0)	11 (78.5)	75 (87.2)
	HTS	4 (6.7)	3 (25.0)	3 (21.5)	10 (11.6)
	MSM and IDU	1 (1.7)			1 (1.2)*
CD4 cell count at diagnosis (cell/mm^3^)	<350	20 (33.3)	7 (58.3)	3 (21.4)	30 (34.9)
	350–500	16 (26.7)	1 (8.3)	6 (42.9)	23 (26.7)
	>500	24 (40.0)	4 (33.3)	5 (35.7)	33 (38.4)
Mean of partners (range)		23.5 (1–360)	8.3 (1–40)	17.4 (2–60)	20.4 (1–360
Number of partners identified		113	18	68	199

Men who have sex with men(MSM), injecting drug use (IDU), and heterosexual (HTS).

### Case-finding effectiveness

**Fig 1:**
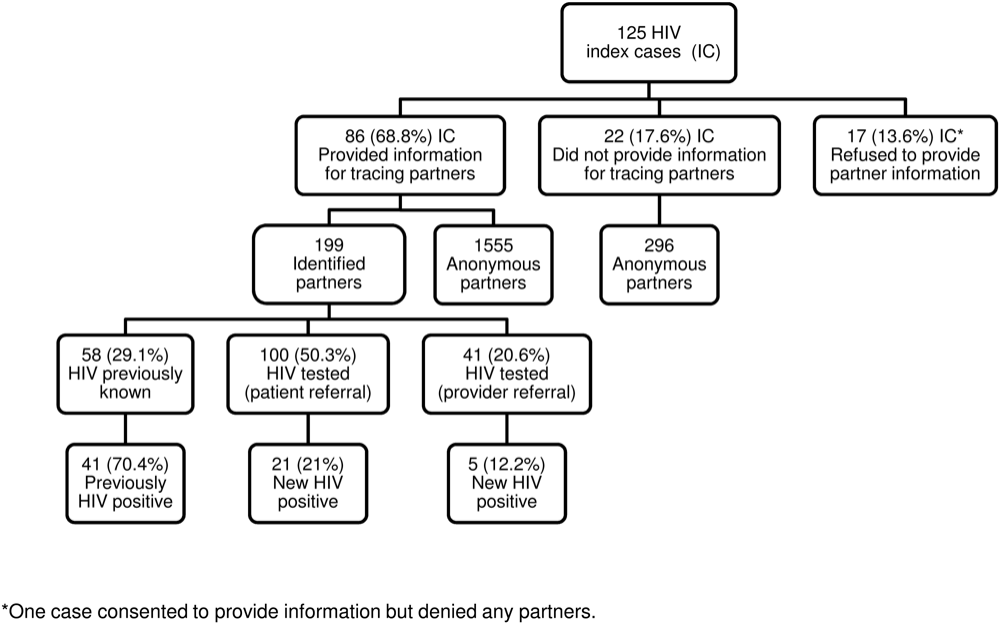
Outcome of partner notification for 125 individuals newly diagnosed with HIV infection. Barcelona, 2012–13.

Fig 1 shows the outline of HIV partner notification activity. Among the 199 identified partners, HIV status was already known in 58, and 70.7% (41/58) of them were previously known to be HIV-positive. Of the remaining 141 partners, 100 were notified by patient referral and agreed to be tested for HIV, and 21.0% (21/100) were newly diagnosed with HIV; 41 partners were notified by provider referral, and 12.2% (5/41) were found to be HIV positive.

The 26 newly identified HIV partners (3 women and 23 men) were sex partners of 24 male index cases (21 MSM, and 3 HTS). Among these index cases four had a CD4 count lower than 200 cells/mm^3^ (one of these met criteria for AIDS), three were between 200–349, five were between 350 and 500 cell/mm^3^, and twelve more than 500 cells/mm^3^.

The total prevalence of HIV among the 199 identified partners was 33.6%. This pilot program reached an overall case-finding effectiveness of 18.4% (95% CI:12.0%-24.8%) among the 141 partners tested as result of the PN program. The NNTI per new HIV infection detected was 4.1 (108/26). The NNTI per new HIV infection detected according to the CD4 of the interviewed index cases was 5.5 (22/4), 6.3 (19/3), 5.2 (23/5), and 2.9 (41/14) for CD4≤200, 201–350, 351–500, and >500 cells/mm^3^, respectively.

**Fig 2:**
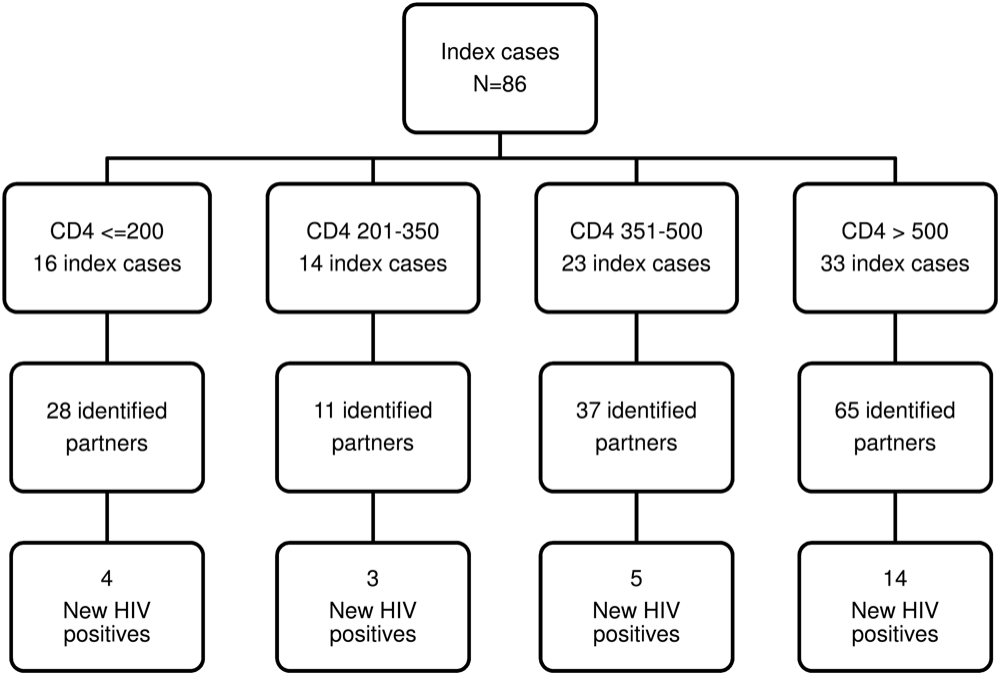
Partner notification outcomes by CD4 count categories of the index cases who provide contact tracing information. Barcelona, 2012–13.

Fig 2 shows the distribution of the 26 new HIV-positive partners according to the CD4 count of the 86 interviewed index cases who provide contact tracing information.

## Discussion

This study provides evidence of the effectiveness of PN in a HIV program implemented jointly by a hospital HIV unit, STI ambulatory clinic, and public health service. This active PN program was feasible, acceptable to the user, and able to identify a high proportion of HIV-infected patients who were unaware of their status.

The acceptability of patient-referral contact tracing ranges from 55% to 97% when clients are asked if they would, hypothetically, be willing to personally disclose to partners if they were diagnosed with HIV [14]. In our study 86.0% of index cases who provided information for tracing partners had disclosed their HIV status to some partners.

Although their interpretation requires caution, our results can be compared and contrasted with those of other PN studies performed in Europe and the United States [7,15]. In these studies, a range of 1 to 8 partners was identified per index case, and the proportion of notified when partners were identified achieved around 70%. The main difference between these studies and ours is the relationship between the percentage of partners notified when they are identified. When information was available, our program was able to contact all partners. We believe that this optimal result was reached because of follow-up used by the public health worker responsible for the PN, and the active involvement of clinicians dealing with cases.

There is considerable variability in the number of contacts identified relative to the total number of contacts surveyed. In a San Francisco-based study [15], this percentage was similar to that observed in our program (11%), whereas in a recent study in the Netherlands it was as high as 36% [16]. The authors of the latter study attributed this high percentage to the possibility that the index cases only reported contacts for whom they had contact details, but not all of their contacts.

There is little evidence regarding which is the most efficient method for PN [9], although some authors suggest that provider referral is more effective [6,17]. We found both the patient and provider referral method to be effective (21% and 12%, respectively). As in other programs, patient referral was the most commonly used method [18]. However, the main drawback of this method is the lack of certainty ensuring that contacts have been informed of their exposure to risk, and in determining whether they have truly been tested, and whether the reported result is real.

Our findings on the prevalence of HIV infection among contacts who were unaware of their exposure (18.4%) are broadly consistent with those observed in diverse studies (20% average) [7,19]. This level of effectiveness is higher than that observed for other interventions such as opt-out HIV testing and outreach programs in our city[20,21,22].

Since almost half (46%) of undiagnosed HIV infections were detected among the partners of index cases with long-standing HIV infection, and since there are more people with long-standing HIV infection than with newly identified HIV infections, these individuals might contribute equally or even more in absolute terms to ongoing HIV transmission than new cases of HIV infection[13,23]. For this reason, we believe that PN should be offered to all individuals who are newly diagnosed with HIV, regardless of their immunological status. In addition to its effectiveness in detecting new cases of HIV, PN offers an excellent opportunity for health education, among both HIV-positive and HIV-negative contacts, for HIV testing to reduce the number of persons who are unaware of their infection, and to reduce late diagnosis.

While not directly applicable to our context, the cost-effectiveness model used in the USA is noteworthy. Under this model, an HIV PN program is cost-effective when one new infection is identified for every≤9.3 index patients interviewed. In our study, we needed to interview 4.8 index cases for every newly detected case of HIV infection [13].

The limitations of this study derive from the impossibility of verifying the results of the HIV test in partners when the index case chose the patient referral method. However, we believe that our results are likely to reflect reality because they do not differ markedly from those observed in other contexts and populations [7]. If identified partners differ from those who were not identified, our result for case-finding effectiveness could not be extrapolated to the rest of the contacts. However, we would argue that the characteristics of index cases are not markedly different from that of cases diagnosed in our city, so we do not believe that this would cause significant biases in our results. However, it is possible that the effectiveness could be underestimated due to the fact that the unidentified contacts were those in whom risky behaviours were more common, such that the probability of transmission could have been higher [24] and the case-effectiveness of the study even greater. In this sense, it would be desirable for such programs to obtain more detailed information about the contacts who are offered the PN.

The main limitation of PN is the high percentage of unidentified sex partners, for the most part because they were anonymous, and in these cases the transmission of HIV has probably been high [25]. This observation makes it necessary to implement and improve programs aimed at this high-risk population. Internet-based PN, outreach programs in non-traditional or non-medical settings, such as bathhouse programs, field visits, social networks programs and GIS-targeted screening, are resources that will need to be developed in our city in the future [26,27,28].

In our experience, the success of PN depends on the collaboration of the index cases in providing information about their contacts. Both the flexibility of the program, as well as the involvement and support of the clinicians who diagnosed and treated cases of HIV/STI were fundamental for the subsequent relationship between the patients and the staff responsible for PN program. The clinician should introduce the user to the advantages of PN, and explain the role of the PHW. This type of program should be integrated into HIV services and STI centres, as it is done for other STIs (e.g. syphilis and gonorrhoea) and other infectious diseases (e.g. tuberculosis and hepatitis). To do this, in addition to specific resources for these programs, directives and clear guidelines are needed for the implementation of efficient PN programs in this area. As mentioned above PN can be an essential tool for controlling HIV transmission, but has been neglected to date, and there are still no official recommendations on implementing HIV PNin Spain.

Finally, none of the index cases reported adverse effects of partner notification.

In summary, this pilot program provides the first evidence of the effectiveness of PN for HIV in Spain. The program was feasible, acceptable tothe user, and successfully identified a high proportion of new HIV-positive cases who were unaware of their infection status. PN programs should be an essential part of HIV Units, STI, and public health services because they are an excellent opportunity for health education, HIV testing and reduction of late diagnosis.
